# High expression of ladinin-1 (LAD1) predicts adverse outcomes: a new candidate docetaxel resistance gene for prostatic cancer (PCa)

**DOI:** 10.1080/21655979.2021.1968647

**Published:** 2021-09-13

**Authors:** Jianping Li, Ziming Wang, Chong Tie

**Affiliations:** Department of Urology, The Second Affiliated Hospital of Xi’an Jiaotong University, China

**Keywords:** LAD1, prostate cancer, docetaxel resistance

## Abstract

Docetaxel resistance is one of the major obstacles that undermine the treatment outcome of PCa. Exploring molecular mechanisms associated with docetaxel resistance could provide insights into the formulation of novel strategies enhancing the efficacy of PCa treatment. Ladinin-1 (LAD1) is an anchoring filament protein in basement membranes, which contributes to the association of the epithelial cells with the underlying mesenchyme. LAD1 has been implicated in the progression of different cancers. However, its role in PCa remains to be investigated. In the present study, we found that LAD1 was highly expressed in docetaxel-resistant PCa cells, while its expression was significantly suppressed in tumor samples after docetaxel treatment. Moreover, the expression level of LAD1 in PCa tissues was significantly higher than that of normal tissue, and high expression level of LAD1 was significantly associated with adverse outcomes of PCa patients. Finally, high expression of LAD1 in PCa tissue was also correlated with the expression level of genes involving in tumor cell proliferation and invasive behaviors. Collectively, our data suggest that LAD1 may serve as a potential prognostic factor in PCa patients.

## 1. Introduction

Prostate cancer (PCa) has become one of the most common cancers, with a high mortality rate in male patients [1]. Androgen deprivation therapy (ADT) serves as an effective control for locally advanced and metastatic PCa [2]. However, PCa frequently progresses even when the amount of testosterone is reduced to very low levels, which eventually leads to castration-resistant PCa (CRPCa) [3]. The anti-mitotic agent docetaxel has been demonstrated to prolong the overall survival (OS) of patients with castration-resistant PCa [4]. However, PCa often relapses even after a combination therapy of docetaxel and ADT due to inherent or acquired docetaxel resistance [5]. Therefore, docetaxel resistance has been recognized as one of the major obstacles that impairs the efficacy of PCa treatment [6]. Identifying the molecular mechanisms associated with docetaxel resistance holds the promise to develop novel strategies targeting docetaxel resistance in PCa patients.

LAD1 (Ladinin-1) is a collagenous anchoring filament protein of basement membrane to maintain the cohesion at the dermal-epidermal junction [7]. It contributes to the stability of the connection between the epithelial layers and the underlying mesenchyme [8]. Apart from its structural role, LAD1 has been implicated in the regulation of mitogenic signaling by acting as an adaptor protein in EGF-induced ERK5 cascade activation [9]. It has been also demonstrated that the expression level of LAD1 predicts the survival of clear cell renal cancer patients [10] and is associated with the metastatic potential of colorectal cancer cells [11]. A comparative proteomic study showed that LAD1 was more abundant in lung adenocarcinoma as compared to normal lung tissue [10]. Similarly, compared with normal tissues, LAD1 expression level was significantly elevated in human thyroid cancers [11]. Besides, LAD1 expression could also predict the prognosis of aggressive breast cancer patients [12]. Although these previous studies highlight the role of LAD1 in the progression of different cancers, the expression pattern and the prognostic value of LAD1 in PCa patients remains to be elucidated.

The wide application of next-generation sequencing technology has revolutionized the biomarker identification for different cancers including PCa, which is of great significance for early therapeutic intervention [13]. To investigate the expression pattern and the prognostic value of LAD1 in PCa patients, we performed transcriptome profiling in docetaxel-sensitive and -resistant PCa cell lines. Furthermore, we also explored the expression pattern of LAD1 in PCa patient sample before and after docetaxel treatment. We further examined the correlation of expression levels between LAD1 and genes involving cell proliferation and invasion in PCa samples. Taken together, our results imply that LAD1 may play a crucial role in docetaxel resistance development and could serve as a potential prognostic factor in docetaxel-resistant PCa patients.

## 2. Materials and methods

### 2.1 Microarray data and analyses of docetaxel-resistant (DR) cell lines versus parental cell lines

Two previously published datasets were used for gene expression profiling analysis of docetaxel-resistant cell lines versus parental cell lines, including GSE158494, GSE33455, both of which can be obtained from NCBI-GEO database (Gene Expression Omnibus) (www.ncbi.nlm.nih.gov/gds/). GSE158494 was based on platform GPL26944 [HuGene-2_0-st] Affymetrix Human Gene 1.0 ST Array. GSE33455 was based on platform GPL570[HG-U133_Plus_2] Affymetrix Human Genome U133 Plus 2.0 Array. In these datasets, Docetaxel-resistant cell lines were generated from PCa cell line models DU-145 and PC-3. DU-145DR and PC-3DR cells acquired levels of resistance to docetaxel that were 1.5 to 2.1 times higher than their parental cells.

GSE158494 datasets was used to identify key molecular genes in docetaxel-resistant cell lines versus original cell lines, genes with a fold change of |logFC |≥1 and false discovery rate (FDR) <0.05 were considered to be differentially expressed. Hierarchical clustering and volcano plot were performed to display the differentially expressed genes (DEGs) [14]. The Venn diagram software was subsequently used to examine the differentially expressed genes (DEGs) shared by two datasets (DU-145-DR PCa cells versus parental cells and PC-3-DR PCa cells versus parental cells) [15]. GO (Gene Ontology) and KEGG (Kyoto Encyclopedia of Genes and Genomes) analyses were performed using the DEGs shared by two datasets, via the Database for Annotation, Visualization and Integrated Discovery (DAVID) v6.8 (david.ncifcrf.gov/). Key GO terms and KEGG pathways were filtered use the cutoff of P < 0.05 and at least two associated genes [15]. To examine the relative LAD1 expression level in DR PCa cells and PCa tissue, expression level of LAD1 was computed as the mean value of all specifically annotated probe sets [16]. All the analyses were performed using the R 4.0.3 software packages.

### 2.2 Microarray data and analyses of PCa tumor tissue and control tissue

Two cohort datasets from NCBI-GEO database and a third cohort dataset from GEPIA2 (Gene Expression Profiling Interactive Analysis 2, gepia2.cancer-pku.cn/, a web server comprising RNA sequencing expression data of 9,736 tumors and 8,587 normal samples from the Cancer Genome Atlas (TCGA) and the Genotype-Tissue Expression (GTEx) projects) were used for gene expression profiling analysis of PCa tissue and control tissues.

The first cohort was derived from GSE134051, which was based on platform GPL26898 Agilent-058029 Custom human expression microarray (Probe Name version). This cohort included 169 PCa patients who underwent radical prostatectomy (RPE) and 39 benign prostate hyperplasia (BPH) patients who underwent surgical treatment. PCa tissue stacks had to contain at least 50% tumor cells on either side of the sections were used for further analyses [17].

The second cohort was derived from GSE51005, which was based on platform GPL11154 Illumina HiSeq 2000 (Homo sapiens) and included 6 patients treated with combined ADT and docetaxel for 6 weeks. Tumor biopsies were taken before and after the treatment. Biopsies were specifically taken from tumor-rich areas of the prostate, where over 60% of the initial diagnostic cores were occupied by tumor [18].

The third cohort dataset was retrieved from GEPIA2, which compiled 492 prostate tumor samples and 52 normal samples for LAD1 expression analysis. Pearson rank correlation coefficient analysis was performed to examine the correlation between LAD1 expression and PCa progression as well as several genes involved in cell proliferation and invasion (including PCNA, CyclinD1, Survivin, N-cadherin, MMP9 and E-cadherin). In addition, PCa patients were divided into high-expression and low-expression groups based on the median value of LAD1 expression in all the patient samples. The Kaplan–Meier method and log-rank test were used to estimate the association between LAD1 expression and overall survival (OS) as well as the disease-free survival (DFS).

The analyses of the first two cohort were performed using the R 4.0.3 software packages. The third cohort analyses were performed in GEPIA2 online database.

### 2.3 Statistical analyses

Statistical significance of LAD1 expression level between two groups (DR cells vs parental PCa cells or PCa tissue vs relative normal tissues) was determined using two-tailed Student’s *t*-test. Pearson rank correlation coefficient analysis was performed to examine the correlation between LAD1 expression level and genes involved in cell proliferation and invasion. *P*-value <0.05 was considered statistically significant and all tests were two sided.

## 3. Results

Prostate cancer has become a profound health threat to male patients worldwide [19]. To identify potential prognostic biomarkers for PCa in response to docetaxel, we retrieved transcriptomic data of prostate tumor samples and normal samples from the Cancer Genome Atlas (TCGA) and the Genotype-Tissue Expression (GTEx) project. We found that LAD1 was upregulated in docetaxel-resistant (DR) PCa cells and PCa tissue. Further analysis showed that high LAD1 expression level was associated with a poorer prognosis of PCa patients. Our study implies that LAD1 may be a useful prognostic indicators or therapeutic targets for PCa.

### 3.1 Identification of differentially expressed genes (DEGs) between DR Cell Lines parental cell lines

**Figure 1.**
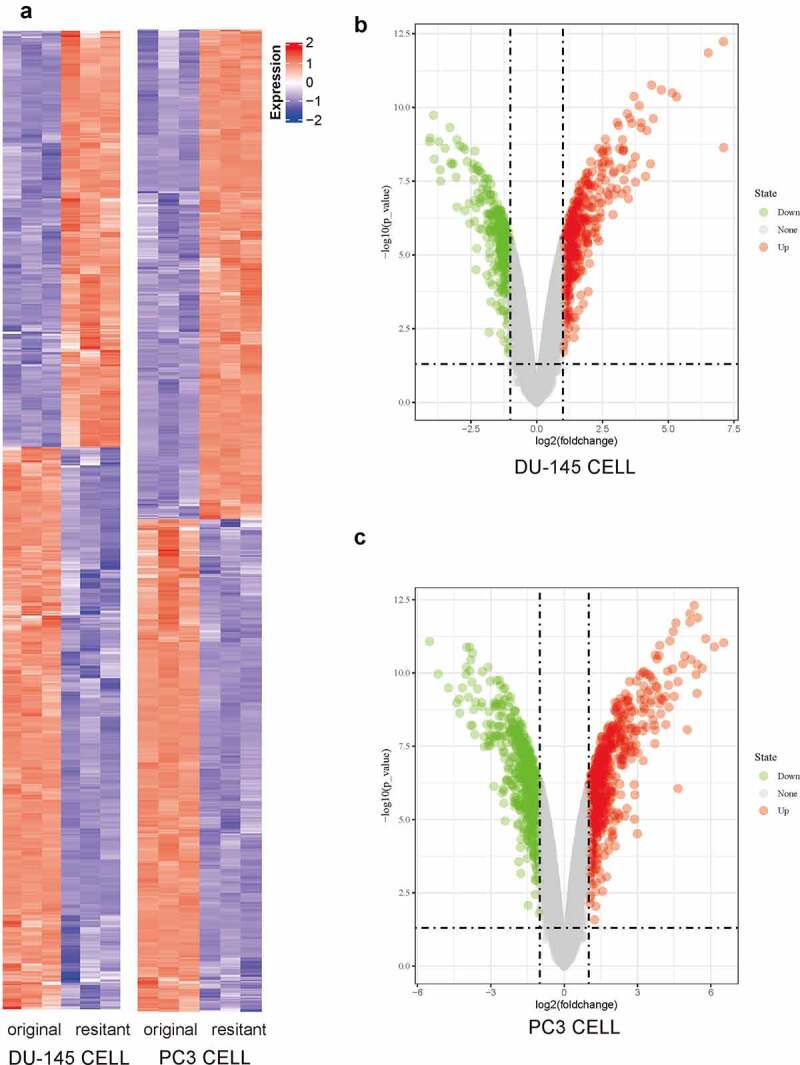
Microarray data were used to compare the transcriptomic changes between original (parental) PCa cells and docetaxel-resistant (DR) PCa cells. (a) Heat map of the DEGs (|logFC| ≥1 and *P*< 0.05) in DU-145-DR and PC-3-DR cells versus parental cells. Upregulated DEGs were shown in red and downregulated DEGs were shown in blue. (b) Volcano plots showing significant DEGs between DU-145-DR cells and original cells. The red and green dots represented the significantly upregulated and downregulated DEGs, respectively (|logFC| ≥1 and *P*< 0.05). (c) Volcano plots showing significant DEGs between PC-3-DR cells and original cells. The red and green dots represented the significantly upregulated and downregulated DEGs, respectively (|logFC| ≥1 and *P* < 0.05)

Using a previously published microarray dataset (GSE158494), we first analyzed the expression profiles of mRNA in two Docetaxel Resistance (DR) Cell Lines (DU-145DR and PC-3DR) and their original (parental) cell lines. Unsupervised hierarchical clustering analysis of the DEG expression levels are shown in a heatmap (Figure 1a). DEGs (|logFC| ≥1 and false discovery rate (FDR) <0.05) were displayed in volcano plots (Figure 1b). The microarray data analysis revealed 814 and 781 DEGs in DU-145-DR and PC-3-DR cells versus the corresponding original cells, respectively.

### 3.2 Functional annotation and pathway enrichment of DR-associated genes

**Figure 2.**
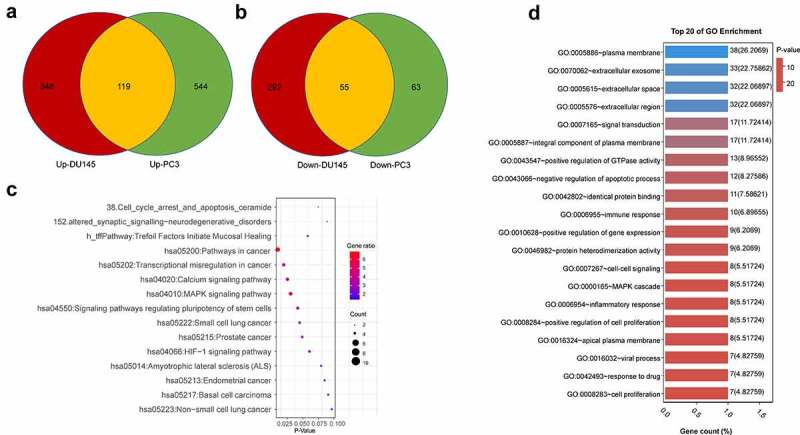
Functional annotation and pathway Enrichment of docetaxel resistance associated genes. (a) Upregulated DEGs shared in both DU145 DR and PC3 DR PCa cells as compared to the original cells. (b) Downregulated DEGs shared in both DU145-DR and PC3-DR PCa cells as compared to the parental cells. (c) Top 20 GO enrichment terms of commonly upregulated and downregulated DEGs. (d) KEGG pathways enrichment terms of commonly upregulated and downregulated DEGs

We next sought to investigate the functional enrichment of the DEGs associated with docetaxel resistance in both PCa cell lines. Venn diagram showed the common DEGs shared by two datasets: a total of 174 common DEGs, including 119 upregulated genes (logFC≥1) and 55 downregulated genes (logFC ≤1) (Figure 2a and b, Table S1). We next performed gene ontology (GO) and KEGG pathway enrichment analysis using Database for Annotation, Visualization and Integrated Discovery (DAVID) v6.8 (https://david.ncifcrf.gov/). KEGG pathway enrichment analysis showed that signaling pathways including pathways in cancer, transcriptional dysregulation in cancer, calcium signaling pathway, and MAPK signaling pathway were overrepresented in that DEGs (Figure 2c). The DEGs related to the top 20 KEGG pathways were showed in Table S2. GO enrichment analysis showed that DEGs were highly enriched in functional clusters including extracellular space and region, extracellular exosome, signal transduction, negative regulation of apoptotic process and positive regulation of GTPase activity (Figure 2d). The DEGs related to the top 20 GO terms were shown in Table S3.

### 3.3 Overexpression of LAD1 in docetaxel-resistant PCa tissues

**Figure 3.**
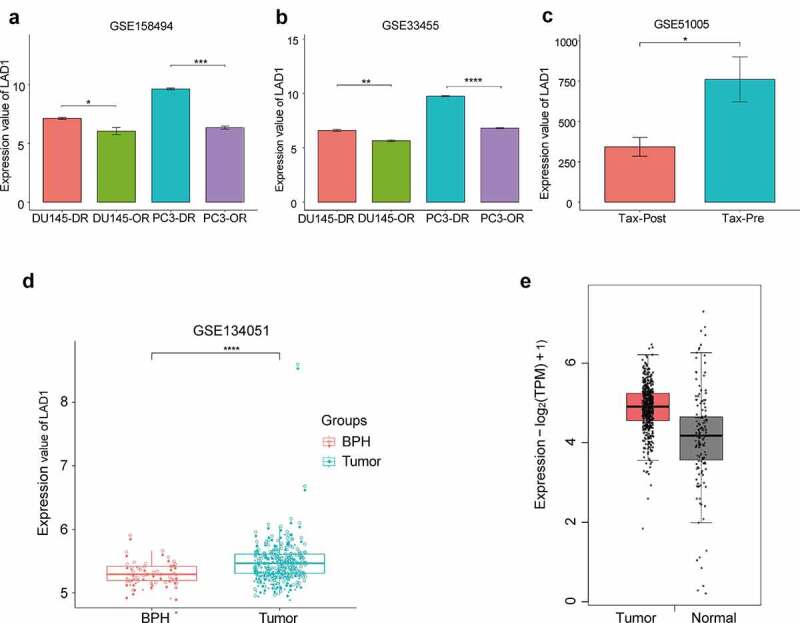
Differential expression analysis of LAD1 in original (OR) PCa cells and docetaxel-resistant (DR) PCa cells, as well as between PCa tumor samples and normal tissues. (a-b) Relative expression of LAD1 between original (OR) PCa cells vs docetaxel-resistant (DR) PCa cells (GSE158494 and GSE33455). (c) Relative expression of LAD1 between PCa tumor tissues before and after combined therapy of ADT and docetaxel. (d) Relative expression of LAD1 between PCa tumor tissues vs benign prostate hyperplasia (BPH) tissues. (e) Relative expression of LAD1 between PCa tumor tissues vs normal tissues (TCGA and GTEx project using GEPIA2 databases). *P < 0.05; **P < 0.01; ***P < 0.005; ****P < 0.001

The 174 common DEGs are shown in Table S1, and LAD1 was one of the most upregulated genes. Many of these top-ranked DEGs have been studied in PCa cells. However, to the best of our knowledge, whether LAD1 expression contributes to PCa progression is unknown. Therefore, we selected LAD1 as a potential target gene for docetaxel resistance development in prostate cancer. As shown in Figure 3a, LAD1 was significantly upregulated in docetaxel-resistant PCa cells (DU-145-DR and PC-3-DR) in comparison to the original PCa cells (DU-145-OR and PC-3-OR). We also analyzed LAD1 expression using another dataset (GSE33455) containing docetaxel-resistant and original PCa cells. In both GSE158494 and GSE33455 datasets, LAD1 expression was significantly upregulated in docetaxel-resistant PCa cells (Figure 3a and b). Additionally, tumor biopsy samples from six PCa patients before and after treatment (combined therapy of ADT and docetaxel for 6 weeks) were analyzed. As we suspect, due these people were non-docetaxel-resistant patients [20], post-treatment samples exhibited a significantly lower LAD1 expression level than pre-treatment samples (Figure 3c, GSE51005). This result further indicates that docetaxel treatment is selected for the cells with high LAD1 expression.

In addition, a higher expression level of LAD1 was observed in PCa tumor samples than BPH (benign prostate hyperplasia) samples (Figures 3d, 164 PCa samples vs 39 BPH samples, GSE134051). We further compared the expression level of LAD1 between PCa tumor tissues and normal tissues from TCGA and GTEx project using GEPIA2 databases. This analysis consistently showed the upregulation of LAD1 in PCa tumor samples (Figures 3e, 492 PCa tissues vs 152 normal tissues, TCGA and GTEx data). Collectively, the above analysis highlighted the potential role of LAD1 upregulation in PCa tumors and its downregulation in the DR PCa cells.

### 3.4 High expression of LAD1 is associated with a poorer prognosis in PCa patients

**Figure 4.**
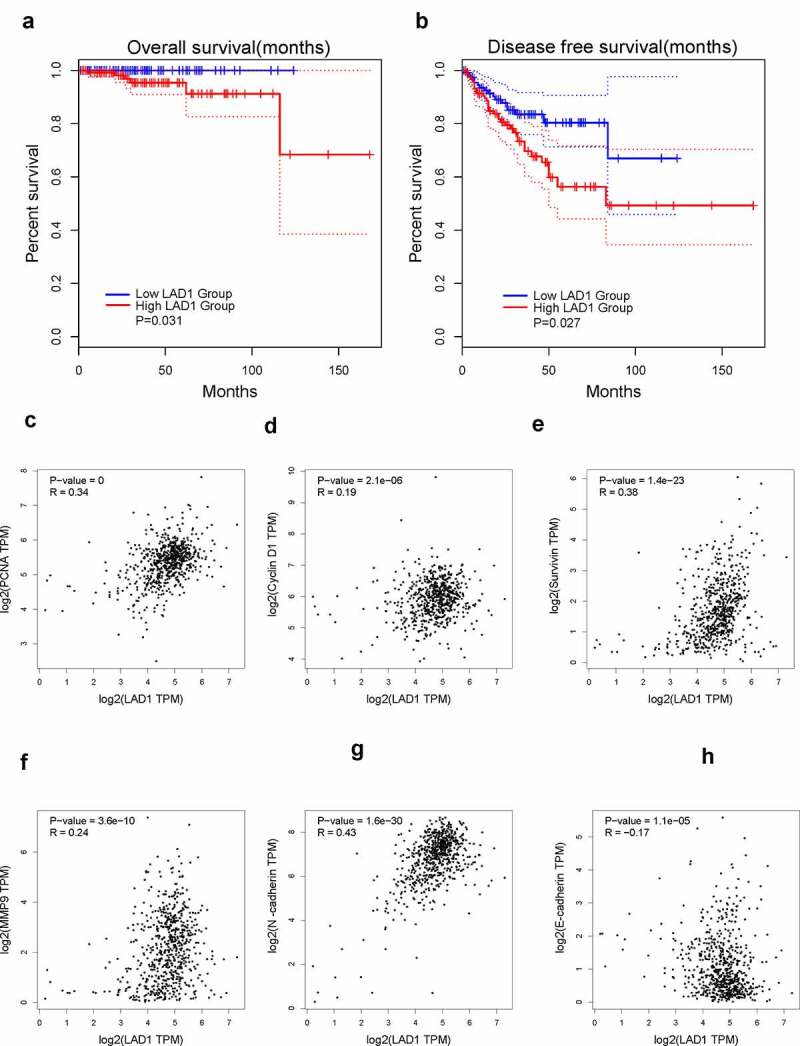
High expression of LAD1 was associated with adverse outcomes in PCa patients. (a) Kaplan–Meier method and log-rank test were performed to analyze the relationship between LAD1 expression level and overall survival (OS). (b) Kaplan–Meier method and log-rank test were performed to analyze the relationship between LAD1 expression level and disease-free survival (DFS). (c–h) Pearson correlation coefficient analysis was performed to examine the correlation between LAD1 expression level and genes involved in cell proliferation and EMT

To investigate the relationship between LAD1 expression level and the overall survival of PCa patients, we retrieved the patient data from GEPIA2 database. PCa patients were divided into high-expression and low-expression groups based on the median value of LAD1 expression in all the patients. The Kaplan–Meier plot based on log-rank test showed that high LAD1 expression level was correlated with a poorer overall survival (OS) (Figure 4a, p = 0.031), as well as a poorer disease-free survival (Figure 4b, p = 0.027). These results indicated that high LAD1 expression level could be used as a potential marker for PCa progression and disease prognosis.

We also analyzed the correlation between LAD1 expression level and several genes involved in cell proliferation and invasion based on the PCa patient data from TCGA database. LAD1 expression exhibited a positive correlation with the expression level of PCNA (*R* = 0.34, *P* = 0.001), CyclinD1 (*R* = 0.19, *P* = 2.1e-06), Survivin (*R* = 0.38, *P* = 1.4e-23), MMP9 (*R* = 0.24, *P* = 3.6e-10) and N-cadherin (*R* = 0.43, *P* = 1.6e-30), while LAD1 expression was negatively correlated with that of E-cadherin (*R* = −0.17, *P* = 1.1e-05). Therefore, high LAD1 expression may also inform the increased expression of genes in cell proliferation and invasion (PCNA, CyclinD1, Survivin, MMP9 and N-cadherin) and the downregulation of epithelial gene (E-cadherin).

## 4. Discussion

Although previous studies have reported many genes to be associated with docetaxel resistance, the pathogenesis of docetaxel resistance in castration-resistant PCa remains unclear [17,18]. In this study, we compared gene expression profiles of two models of docetaxel resistance and castration-resistant PCa cell lines, and investigated the potential genes involved in docetaxel resistance. We found 174 DEGs that were consistently deregulated in both DU-145 and PC-3 R DR-PCa cell lines, as compared to their parent cell lines. Consistent with previous studies, most of the DEGs (such as IGFBP3 [19], ITGB3 [21], ZEB1 [22], GJA1 [23], MMP14 [24], GLI2 [25], AREG [26], LCN2 [27], MACC1 [28], GALNT3 [29] and NCOA1 [30]) were involved in a wide spectrum of processes in tumorigenesis and tumor progression, such as the self-renewal of cancer stem cell, the invasion and metastasis. Interestingly, many of these DEGs (such as IGFBP3 [31], ITGB3 [32], ZEB1 [33], GJA1 [34], MMP14 [35], GLI2 [36] and NCOA1 [37]) were previously reported to contribute to PCa progression. It is worth noting that certain genes (such as IGFBP3 [38], GJA1 [39]) have been implicated in docetaxel treatment efficacy.

Functional enrichment analysis such as KEGG analysis revealed that several pathways in cancer, including transcriptional dysregulation in cancer, calcium signaling pathway and MAPK signaling pathway, were overrepresented in the DEGs between DR PCa cell line and the parental cell lines. This indicates that calcium and MAPK signaling pathway alteration may contribute to the development of docetaxel resistance. It has been reported that taxanes-induced drug resistance is accompanied by the dysregulation of signaling pathways which promote cancer cell survival and cell growth [40]. For example, CDH1 is a key molecule in calcium signaling pathway, and Wang et al. reported that CDH1 is significantly downregulated in docetaxel-resistant prostate cancer cells [18]. In addition, the activation of MAPK signaling was also reported to contribute to docetaxel resistance development in MCF-7 cells [41]. Our analysis adds novel evidence that altered calcium signaling and MAPK signaling could contribute to docetaxel resistance. The analysis of prostate cancer cell lines identified potential genes involved in docetaxel resistance in castration-resistant PCa. Further clinical sample validation is needed to confirm the roles of these genes and signaling pathways in patients with castration-resistant PCa.

The majority of DEGs have been reported in PCa cells or docetaxel resistance. However, the role of LAD1 on PCa progression and docetaxel-resistance was unclear. Our analysis showed that LAD1 expression was upregulated in docetaxel-resistant cells in comparison to the parental cells, suggesting that LAD1 upregulation may contribute to docetaxel resistance development in PCa. Transcriptome analysis on PCa biopsy tumor samples before and after combined therapy of docetaxel and ADT further supports the downregulation of LAD1 after docetaxel treatment. However, future studies are required to investigate the underlying mechanisms by which LAD1 overexpression contributes to docetaxel resistance. We also compared the expression of LAD1 in PCa tumor tissue and normal tissue, and found that LAD1 expression level in PCa tumor was significantly higher than that in normal tissue. Survival analysis in the patient indicates that high LAD1 expression was correlated with adverse outcomes of PCa patients, which suggest that LAD1 expression may contribute to the aggressiveness of PCa in patients.

High LAD1 expression may contribute to the aggressiveness of PCa by regulating cells in cell proliferation and invasion, since high LAD1 expression is correlated with the increased expression of genes in cell proliferation and invasion (PCNA, CyclinD1, Survivin, MMP9 and N-cadherin) and the downregulation of epithelial gene (E-cadherin). PCNA is an essential factor for DNA replication in the cell nucleus to support cell proliferation [42], and CyclinD1 is a positive regulator of cell cycle progression, which forms a complex with CDK4 and CDK6 to promote G1-S transition [43]. Survivin is an anti-apoptotic protein, which inhibits apoptotic executioner Caspases [44]. Future studies are required to clarify how high level of LAD1 expression positively regulate these proliferative and pro-survival genes. Epithelial-mesenchymal transition (EMT) is recognized as a major event for metastasis transformation of cancer cells [45]. The downregulation of epithelial marker such as E-cadherin and the upregulation of mesenchymal phenotype marker N-cadherin are key features in EMT [46]. In addition, the invasion of tumor cells to local tissues requires the remodeling of extracellular matrix by proteases such as MMP9 [47]. Our data showed that the expression level of LAD1 in PCa patient samples was positively correlated with that of N-cadherin and MMP9, while it was negatively correlated with the expression of E-cadherin. These data suggest that LAD1 expression level also informs the EMT status of PCa cells and the underlying mechanisms remain to be elucidated.

## 5. Conclusion

In summary, our study provides novel insights into the involvement of LAD1 in docetaxel resistance in PCa. We also showed the potential role of LAD1 as a prognostic biomarker in PCa patients, which is positively correlated with genes involved in cell proliferation, survival and EMT.

## Supplementary Material

Supplemental Material

## Highlights

(1) LAD1 (Ladinin-1) was upregulated in prostatic cancer (PCa) tissue.

(2) LAD1 was upregulated in docetaxel-resistant PCa cells.

(3) High expression of LAD1 was associated with adverse outcomes in PCa patients.
