# Supplementary material for: High expression of ladinin-1 (LAD1) predicts adverse outcomes: a new candidate docetaxel resistance gene for prostatic cancer (PCa)
Source: Bioengineered. 2021 Sep 13;12(1):5749–59. doi: 10.1080/21655979.2021.1968647 (PMC8806705; doi:10.1080/21655979.2021.1968647)
Supplement: Supplemental Material [file KBIE_A_1968647_SM2293.zip › suppl/Table S1.docx]

**Table S1 Common DEGs in both DU145 and PC3 parental cells compared to DR PCa cells.**

| Down-regulated genes |  | DU145-DR vs DU-45 | | PC3-DR vs PC3 | |
| --- | --- | --- | --- | --- | --- |
| Tag | gene symbol | logFC | P.Value | logFC | P.Value |
| 100507032_at | SH2D3A | -1.350781121 | 3.04446E-07 | -1.265318641 | 3.11089E-07 |
| 101559451_at | ADGRG2 | -1.052193844 | 8.99476E-06 | -1.023880998 | 1.56608E-05 |
| 102724515_at | LOC102723373 | -1.808212618 | 0.004197222 | -3.028785836 | 3.05704E-08 |
| 10449_at | CDS1 | -1.632519728 | 3.50297E-07 | -1.040432389 | 4.54129E-06 |
| 10610_at | SPC42 | -1.714821649 | 4.56876E-07 | -1.872766346 | 1.1412E-08 |
| 107985343_at | LOC107984034 | -1.489962955 | 1.50794E-06 | -1.033171029 | 0.000143068 |
| 11201_at | PFD1 | -1.254410436 | 2.43298E-05 | -1.126839149 | 1.97878E-05 |
| 1123_at | GHRHR | -1.092261838 | 1.74896E-05 | -1.004939066 | 4.69768E-06 |
| 1152_at | mal2.L | -1.669649639 | 2.50877E-06 | -1.195972126 | 5.82421E-07 |
| 118429_at | CKB | -1.202737287 | 2.10895E-06 | -1.39996619 | 3.06207E-07 |
| 1200_at | GLI2 | -1.062500321 | 0.000266559 | -1.128723289 | 5.43855E-07 |
| 1396_at | IGFBP5 | -1.36666826 | 0.000103192 | -1.331778052 | 2.52335E-06 |
| 171586_at | C130071C03Rik | -1.365618373 | 3.94373E-07 | -1.600178109 | 2.579E-06 |
| 19_at | CKB | -1.081965197 | 5.06423E-05 | -1.479227227 | 5.15483E-08 |
| 2260_at | PRR15 | -1.193959674 | 9.83754E-07 | -2.236979802 | 1.14491E-07 |
| 22795_at | FGFR1 | -3.025735493 | 2.36779E-07 | -1.516961759 | 6.34632E-06 |
| 22979_at | NID2 | -2.173319468 | 1.27294E-06 | -2.203590375 | 3.30337E-09 |
| 23208_at | EFR3B | -1.056131444 | 4.862E-06 | -1.593450366 | 2.12946E-07 |
| 23331_at | WSCD1 | -1.295827158 | 1.12654E-05 | -1.873705558 | 4.01759E-07 |
| 23708_at | TTC28 | -1.231799758 | 2.56353E-05 | -1.599033331 | 1.13777E-06 |
| 25861_at | GSPT2 | -1.070204377 | 3.50023E-06 | -1.144669543 | 2.35984E-06 |
| 283537_at | SMPDL3B | -1.39016204 | 9.71395E-07 | -2.266484891 | 3.57517E-09 |
| 283554_at | SLC46A3 | -1.322320674 | 2.07208E-06 | -1.404021562 | 4.85964E-07 |
| 30846_at | HAS2 | -1.077744353 | 1.30971E-05 | -1.769567127 | 4.77132E-05 |
| 316_at | EHD2 | -1.395029443 | 6.93468E-07 | -1.584391048 | 1.65318E-08 |
| 3486_at | AMIGO2 | -2.061282035 | 5.82102E-08 | -1.409988904 | 3.94615E-06 |
| 3690_at | IRF6 | -2.724335522 | 2.66329E-09 | -2.748860046 | 1.32281E-08 |
| 389289_at | TMEM238 | -1.263330886 | 1.39014E-06 | -1.145151023 | 2.74396E-05 |
| 4052_at | SAMD12 | -1.077454717 | 5.42899E-06 | -2.207324395 | 9.77825E-10 |
| 4325_at | MMP14 | -1.213476472 | 1.59622E-05 | -1.552890676 | 2.53031E-07 |
| 439921_at | MMP16 | -1.345387379 | 3.35263E-07 | -1.787762183 | 1.49885E-08 |
| 440482_at | MXRA7 | -1.266345418 | 0.000785971 | -1.637323737 | 3.23152E-07 |
| 493_at | CSNK1D | -1.636137586 | 9.61342E-08 | -1.252789671 | 3.15969E-07 |
| 51200_at | C19orf60 | -2.565580969 | 1.80589E-07 | -1.604564079 | 4.20897E-07 |
| 5140_at | TAOK3 | -1.042598922 | 3.05172E-05 | -1.240970278 | 3.55785E-06 |
| 5289_at | SERPINF1 | -1.825859446 | 2.69637E-08 | -1.033032399 | 5.17425E-06 |
| 5327_at | MET10 | -1.24453447 | 5.84057E-06 | -1.053023173 | 1.75936E-06 |
| 54800_at | MANSC1 | -1.10407834 | 0.000104942 | -1.498014454 | 2.26991E-05 |
| 55714_at | LINC00261 | -1.585269464 | 6.67502E-07 | -1.230401599 | 2.82632E-07 |
| 5654_at | SER3 | -1.47956832 | 2.54631E-05 | -1.929404128 | 3.69303E-09 |
| 57088_at | JPH1 | -1.01807898 | 7.71098E-05 | -1.715935511 | 7.63092E-08 |
| 57636_at | SFMBT2 | -1.221528107 | 1.34236E-05 | -1.877706993 | 2.76381E-06 |
| 5797_at | SFMBT2 | -2.099558386 | 1.66944E-08 | -1.51081845 | 4.43918E-08 |
| 59277_at | PANK2 | -1.060757752 | 8.74651E-06 | -1.469804203 | 8.19815E-06 |
| 64175_at | IFIH1 | -1.358333866 | 4.36407E-06 | -1.477546276 | 1.5408E-06 |
| 65250_at | FRG1JP | -1.188898479 | 9.11772E-05 | -1.896581347 | 4.70099E-08 |
| 654_at | MXI1 | -1.19002829 | 9.32175E-07 | -1.308585661 | 2.32641E-05 |
| 677769_at | ST14 | -1.398714339 | 6.50929E-07 | -1.903912606 | 1.5779E-08 |
| 6935_at | YCL042W | -1.027072749 | 7.12284E-05 | -2.669729444 | 7.18761E-10 |
| 7041_at | ZEB1 | -1.794342574 | 8.60553E-06 | -1.708808425 | 1.3633E-06 |
| 79901_at | ARHGAP28 | -1.478260843 | 1.05486E-06 | -3.071756126 | 1.14817E-08 |
| 83478_at | RSRC1 | -1.682826726 | 5.65701E-07 | -1.202272558 | 4.82064E-07 |
| 84911_at | IRX5 | -1.681713052 | 1.0679E-06 | -1.787203252 | 8.92334E-05 |
| 8506_at | EXO1 | -1.506265913 | 8.23596E-07 | -1.442049172 | 3.0525E-07 |
| 85360_at | CNTNAP2 | -1.044138022 | 1.31313E-05 | -1.673189094 | 3.94339E-08 |

| Up-regulated genes |  | DU145-DR vs DU-145 | | PC3-DR vs PC3 | |
| --- | --- | --- | --- | --- | --- |
| Tag | gene symbol | logFC | P.Value | logFC | P.Value |
| 100418865_at | LINC00941 | 1.359134853 | 5.95964E-05 | 1.127199448 | 0.000196938 |
| 10045_AT | CTS1 | 1.234667593 | 1.25251E-05 | 1.878066019 | 2.17802E-06 |
| 101927204_at | AASS | 1.062326993 | 9.53464E-05 | 1.454354935 | 1.75994E-06 |
| 10205_AT | ACE2 | 3.969064247 | 4.86351E-10 | 5.439115633 | 5.07848E-11 |
| 102723373_at | MPZL2 | 1.954619318 | 4.10494E-06 | 2.88431954 | 7.5644E-09 |
| 1040_at | LINC01291 | 1.753011258 | 1.01968E-07 | 3.203576502 | 9.83446E-11 |
| 105370503_at | ACAA2 | 1.606081727 | 3.91637E-05 | 1.535503329 | 1.24753E-07 |
| 10602_AT | YKL050C | 1.057036055 | 5.78304E-06 | 1.50812764 | 2.75382E-07 |
| 10628_AT | NUP100 | 1.526494476 | 5.82719E-06 | 3.234386302 | 9.83003E-08 |
| 107984034_at | TXNIP | 1.195157827 | 0.001029549 | 1.35223239 | 0.00032949 |
| 107986700_at | LINC01836 | 1.358667998 | 7.65265E-07 | 1.339003552 | 1.54982E-05 |
| 11001_at | heparanase | 1.386398841 | 1.02041E-06 | 4.152387391 | 5.72507E-09 |
| 11240_at | CHN1 | 2.618154259 | 2.93728E-08 | 1.080923663 | 3.04458E-06 |
| 114569_AT | Kmt5b | 7.097576073 | 5.89316E-13 | 1.008377233 | 7.49983E-07 |
| 120224_at | TPP1 | 1.801246643 | 1.14786E-07 | 3.530661132 | 2.36599E-09 |
| 121504_at | TMEM45B | 1.069001791 | 5.81971E-05 | 1.407614673 | 1.00613E-07 |
| 121512_at | H4C6 | 1.102122659 | 3.89968E-06 | 1.094719477 | 5.86723E-05 |
| 123036_at | FGD4 | 4.137851596 | 2.1838E-08 | 5.189123931 | 3.51243E-11 |
| 130340_AT | Speer8-ps1 | 1.112847035 | 1.31272E-05 | 1.030723156 | 3.37663E-06 |
| 1366_at | NCOA1 | 1.455620993 | 1.98007E-06 | 3.796471595 | 3.36312E-11 |
| 1438_AT | EPHB3 | 1.487315443 | 5.42661E-05 | 1.082803132 | 8.90478E-06 |
| 150590_AT | MED28 | 1.42083388 | 4.89662E-05 | 2.844097155 | 1.40179E-08 |
| 152519_AT | nemy | 1.446589557 | 1.19837E-05 | 1.657255354 | 7.24047E-07 |
| 153562_AT | CG7280 | 1.481435664 | 2.94871E-07 | 2.381119662 | 3.89406E-09 |
| 157638_at | MARVELD2 | 1.990942512 | 7.14019E-07 | 3.90677659 | 1.15537E-11 |
| 220_at | ALDH2 | 1.078886905 | 1.52046E-05 | 1.382461737 | 1.21743E-07 |
| 2202_at | ALDH1A3 | 1.42253868 | 1.54253E-07 | 5.31555881 | 4.94465E-13 |
| 221806_at | EFEMP1 | 2.580227392 | 6.61264E-09 | 1.121090473 | 4.17182E-07 |
| 222171_at | VWDE | 1.046438757 | 2.75553E-05 | 2.430265763 | 1.76287E-08 |
| 23240_at | SYTL2 | 1.261881709 | 2.08874E-06 | 1.323431194 | 2.93138E-06 |
| 23302_at | TMEM131L | 1.427970686 | 4.37049E-07 | 1.773954746 | 8.20678E-09 |
| 2591_at | WHRN | 1.692769716 | 1.28762E-06 | 1.95162306 | 6.98517E-09 |
| 26002_at | GALNT3 | 1.111187213 | 0.000154953 | 2.491300512 | 3.40932E-08 |
| 26353_at | CCDC69 | 1.232403636 | 0.000256695 | 1.239056635 | 1.77506E-05 |
| 2697_at | HSPB8 | 2.748233039 | 6.71628E-09 | 1.043644176 | 1.38697E-05 |
| 2706_at | GJA1 | 1.82336639 | 5.60184E-07 | 4.654097881 | 8.78455E-07 |
| 27293_at | ANKRD1 | 1.575544173 | 1.77157E-07 | 2.279219239 | 1.59998E-08 |
| 288_at | GPR137C | 1.625039367 | 4.42388E-08 | 1.019912902 | 1.71227E-06 |
| 29126_at | ANK3 | 2.522603808 | 2.9933E-09 | 2.276091989 | 7.25928E-08 |
| 2983_at | GRPR | 1.706978193 | 3.55982E-08 | 1.487485487 | 6.51737E-07 |
| 29842_at | GUCY1B1 | 1.120020679 | 7.80718E-06 | 1.286823634 | 2.59419E-06 |
| 3219_at | FOXA2 | 1.660427433 | 6.88182E-08 | 1.998742681 | 3.38273E-05 |
| 3224_at | HOXB9 | 1.10011607 | 3.37926E-06 | 1.500148254 | 2.72104E-07 |
| 3428_at | Igsf3 | 1.104780743 | 1.02964E-05 | 1.089294405 | 1.63046E-05 |
| 344462_at | IFI16 | 1.545629993 | 3.86167E-06 | 1.391107821 | 2.34044E-07 |
| 346389_at | KRT18P39 | 1.109189947 | 0.002415119 | 3.707524549 | 2.57584E-08 |
| 347735_at | MACC1 | 1.109594125 | 1.08051E-05 | 1.103294759 | 5.64113E-07 |
| 347902_at | SERINC2 | 1.174158351 | 5.70188E-06 | 1.607807749 | 8.78952E-05 |
| 360019_at | IGFBP3 | 2.361232886 | 3.42521E-06 | 1.591323826 | 2.74038E-06 |
| 3664_at | KRT18P10 | 1.423328532 | 8.37166E-06 | 2.714996204 | 1.40978E-09 |
| 374_at | ITGB3 | 3.699873416 | 4.23328E-11 | 3.498490542 | 3.8471E-10 |
| 374393_at | AREG | 1.048168651 | 0.000125073 | 1.661821212 | 1.09826E-05 |
| 388564_at | PEAR1 | 1.021586673 | 0.000201463 | 1.012205756 | 1.92995E-06 |
| 3898_at | ANXA2R | 1.612403249 | 1.72669E-05 | 3.263926001 | 8.90637E-11 |
| 3934_at | LAD1 | 1.085538225 | 0.001148091 | 3.284397083 | 1.6821E-09 |
| 401474_at | LCN2 | 1.046088014 | 9.23804E-05 | 1.260507865 | 4.4094E-07 |
| 4070_AT | LYS1 | 3.755196456 | 3.42602E-08 | 4.265746105 | 2.61447E-10 |
| 4072_AT | IRC24 | 4.368848692 | 1.74566E-11 | 4.323144179 | 8.0775E-12 |
| 4602_at | ANKRD20A5P | 1.065697261 | 1.21683E-05 | 1.363797473 | 3.20329E-06 |
| 51347_at | PLAC8 | 1.60201612 | 6.65589E-08 | 1.203895812 | 2.17473E-06 |
| 5265_AT | PDE1 | 2.422144649 | 3.61671E-07 | 2.718375835 | 4.32173E-08 |
| 5321_AT | SAP155 | 1.721385835 | 6.32399E-07 | 1.180944032 | 2.42213E-05 |
| 53836_at | PLAT | 3.192928768 | 1.13821E-08 | 2.203767378 | 3.65222E-09 |
| 54101_AT | XCR1 | 1.29631514 | 3.65508E-05 | 1.439408105 | 1.05101E-07 |
| 54502_AT | PTPRE | 1.061085462 | 9.85755E-06 | 1.683205268 | 1.42636E-06 |
| 54541_at | RBM47 | 2.507621662 | 1.08233E-08 | 2.891090567 | 1.44236E-06 |
| 54682_AT | RPL22L1 | 1.103014651 | 6.81513E-05 | 1.345118766 | 3.23737E-07 |
| 54836_AT | RNF207 | 1.323228343 | 5.77379E-07 | 1.891070476 | 1.0051E-06 |
| 54845_AT | N4BP2 | 5.158311215 | 3.21219E-11 | 6.5213735 | 9.37014E-12 |
| 54894_AT | SNORA11D | 2.128373695 | 2.47757E-07 | 1.671251456 | 6.32677E-06 |
| 55329_AT | TF | 1.402941038 | 1.55084E-06 | 1.974722426 | 3.58778E-08 |
| 55796_AT | SCN8A | 1.132603114 | 3.0211E-05 | 2.587546743 | 8.52142E-10 |
| 5603_AT | AVT6 | 1.55652289 | 1.52508E-07 | 2.155322236 | 1.44552E-09 |
| 56704_at | HTRA1 | 2.418082942 | 5.50501E-07 | 2.670679062 | 3.4616E-09 |
| 57194_AT | PRICKLE2 | 1.21581033 | 9.93595E-06 | 1.549863869 | 3.48952E-07 |
| 5738_at | ATP10A | 1.151146794 | 6.96521E-06 | 4.282033141 | 1.48151E-09 |
| 57480_at | STRIP2 | 1.459275716 | 8.10984E-06 | 2.479168192 | 7.52908E-06 |
| 57530_at | PLEKHG1 | 1.669315921 | 5.89466E-07 | 1.194002994 | 6.94002E-06 |
| 57713_AT | PIGK | 1.292609222 | 1.15338E-06 | 1.367664953 | 3.95591E-07 |
| 58191_at | PTPRM | 1.234352842 | 3.83436E-06 | 1.439212423 | 4.20267E-08 |
| 58489_AT | TMEM120B | 1.621659437 | 2.32725E-06 | 1.558024977 | 1.04445E-07 |
| 586_at | ABHD17C | 1.419420914 | 8.39501E-06 | 1.582477943 | 1.67422E-06 |
| 6196_AT | PMP3 | 1.210612963 | 0.000142735 | 1.499427192 | 6.33567E-07 |
| 6304_AT | HOM2 | 1.035847768 | 0.000544749 | 1.314925181 | 7.83854E-06 |
| 6335_AT | MKC7 | 1.947930348 | 3.80144E-06 | 2.273652742 | 6.18427E-07 |
| 63901_at | SDC2 | 1.152052291 | 3.25453E-06 | 1.606621758 | 6.70498E-08 |
| 64129_AT | AXIN2 | 1.540230078 | 8.93126E-06 | 2.327058025 | 9.08274E-08 |
| 64135_at | TINAGL1 | 1.829702386 | 7.5796E-07 | 1.823308332 | 3.30023E-07 |
| 64284_at | P3H1 | 1.759460587 | 2.66634E-06 | 1.102983584 | 1.95331E-05 |
| 64761_at | RAB17 | 1.228530087 | 6.10725E-06 | 1.127618755 | 2.74333E-07 |
| 6507_AT | FAD1 | 2.34703309 | 3.4933E-08 | 3.895705135 | 1.12426E-09 |
| 652_at | SLC1A3 | 2.507832872 | 7.63892E-09 | 2.383917772 | 8.38229E-08 |
| 6692_AT | RRI1 | 3.303488175 | 2.9743E-09 | 2.535537222 | 8.999E-10 |
| 6768_at | SPINT1 | 3.318863821 | 2.88258E-08 | 4.655071339 | 1.99336E-10 |
| 6850_AT | SRD1 | 2.118623702 | 1.44599E-08 | 2.38622633 | 9.31024E-08 |
| 7164_AT | DUR1,2 | 1.032791846 | 5.90571E-06 | 1.059586927 | 1.95806E-06 |
| 723788_at | TPD52L1 | 2.463643388 | 1.72946E-07 | 3.637562707 | 1.81809E-10 |
| 730755_at | MIG7 | 1.784220826 | 7.25443E-07 | 1.52865112 | 1.64873E-05 |
| 7421_AT | ILS1 | 1.621646335 | 2.43279E-07 | 1.756705686 | 5.94386E-08 |
| 7718_at | ZNF165 | 1.22195736 | 1.14666E-05 | 2.431899407 | 2.99114E-09 |
| 79056_at | ZNF165 | 1.249885229 | 1.26166E-05 | 3.368505558 | 1.10009E-10 |
| 79098_at | PRRG4 | 2.774244423 | 8.34291E-09 | 2.436838499 | 1.40049E-07 |
| 79152_AT | SMN1 | 1.359762811 | 5.86827E-05 | 1.18397667 | 1.45378E-06 |
| 79822_at | TFPI2 | 1.109424537 | 1.76867E-05 | 1.148851102 | 5.72E-05 |
| 81706_AT | AHNAK | 1.173319647 | 1.89949E-05 | 1.091088086 | 1.08684E-05 |
| 84057_at | H3C10 | 1.441548053 | 1.99615E-07 | 1.143279196 | 1.17705E-06 |
| 84561_at | MND1 | 1.313299887 | 4.1963E-06 | 1.372026817 | 1.47709E-07 |
| 84740_AT | FAM78B | 5.325697874 | 4.37827E-11 | 1.213530596 | 6.69338E-05 |
| 84951_at | ZNF382 | 3.447064176 | 1.62512E-10 | 2.587010923 | 5.71717E-09 |
| 84983_AT | P4HA3 | 1.543720765 | 6.35871E-05 | 1.039638927 | 9.88148E-06 |
| 8673_AT | PAP2 | 2.481051823 | 1.67175E-08 | 1.393217073 | 4.38415E-07 |
| 8781_AT | BSC5 | 1.116017226 | 1.67106E-06 | 2.351004352 | 1.29175E-07 |
| 9053_AT | SPS18 | 1.952560723 | 2.07322E-07 | 2.225875424 | 1.27791E-09 |
| 91862_at | CMTM3 | 1.685777242 | 1.14756E-07 | 2.46058205 | 1.75791E-09 |
| 92359_AT | Blk | 1.018658092 | 0.000395917 | 1.53022732 | 2.67224E-06 |
| 93664_AT | Atp1b2 | 1.068441078 | 6.9106E-06 | 1.052070068 | 1.72648E-05 |
| 9723_AT | WAR1 | 1.796570872 | 6.04213E-07 | 1.518384621 | 1.84642E-06 |
| 9914_AT | ATG17 | 1.044759839 | 9.73185E-05 | 3.189588494 | 2.20959E-09 |
| 999_AT | CYP27A1 | 3.969807174 | 3.31591E-10 | 5.135924303 | 1.86917E-12 |
