# Supplementary material for: High expression of ladinin-1 (LAD1) predicts adverse outcomes: a new candidate docetaxel resistance gene for prostatic cancer (PCa)
Source: Bioengineered. 2021 Sep 13;12(1):5749–59. doi: 10.1080/21655979.2021.1968647 (PMC8806705; doi:10.1080/21655979.2021.1968647)
Supplement: Supplemental Material [file KBIE_A_1968647_SM2293.zip › suppl/Table S2.docx]

| KEEG PATHWAY | Count | P-Value | Gene |
| --- | --- | --- | --- |
| 38.Cell_cycle_arrest_and_apoptosis_ceramide | 2 | 0.076840693 | RAB17/MMP14 |
| 152.altered_synaptic_signalling−neurodegenerative_disorders | 2 | 0.008671081 | ANK3/SCN8A |
| Pathway:Trefoil Factors Initiate Mucosal Healing | 4 | 0.004903531 | ITGB3/RAB17/MMP14/SERPINF1 |
| hsa05200:Pathways in cancer | 8 | 0.053712203 | EFEMP1/LAD1/HTRA1/SDC2/TINAGL1/P3H1/NID2/MXRA7 |
| hsa05202:Transcriptional misregulation in cancer | 6 | 0.000062392 | TPP1/SYTL2/ITGB3/RAB17/MMP14/SERPINF1 |
| hsa04020:Calcium signaling pathway | 5 | 0.02109994 | SYTL2/ITGB3/SMN1/AHNAK/LAD1 |
| hsa04010:MAPK signaling pathway | 6 | 0.023336447 | MND1/FAM78B/ZNF382/P4HA3/PAP2/LAD1 |
| hsa04550:Signaling pathways regulating pluripotency of stem cells | 4 | 0.003762885 | EFEMP1/SPS18/HTRA1/SDC2 |
| hsa05222:Small cell lung cancer | 3 | 0.005089123 | TPP1/SYTL2/ITGB3 |
| hsa05215:Prostate cancer | 3 | 0.062214957 | RAB17/MMP14/SERPINF1 |
| hsa04066:HIF−1 signaling pathway | 3 | 0.084977639 | ANK3/SCN8A/SMN1 |
| hsa05014:Amyotrophic lateral sclerosis (ALS) | 3 | 0.08055486 | TPP1/SYTL2/ITGB3 |
| hsa05014:Amyotrophic lateral sclerosis (ALS) | 3 | 0.087835763 | SDC2/TINAGL1/P3H1 |
| hsa05217:Basal cell carcinoma | 3 | 0.088044145 | ANKRD1/ANK3/SCN8A |
| hsa05223:Non−small cell lung cancer | 3 | 0.09209926 | SMN1/AHNAK/JPH1 |

**Table S2 DEGs related to main KEGG enriched pathways**
