# Supplementary material for: High expression of ladinin-1 (LAD1) predicts adverse outcomes: a new candidate docetaxel resistance gene for prostatic cancer (PCa)
Source: Bioengineered. 2021 Sep 13;12(1):5749–59. doi: 10.1080/21655979.2021.1968647 (PMC8806705; doi:10.1080/21655979.2021.1968647)
Supplement: Supplemental Material [file KBIE_A_1968647_SM2293.zip › suppl/Table S3.docx]

**Table S3 DEGs related to top 20 of GO terms.**

| Term | Count | PValue | Gene |
| --- | --- | --- | --- |
| GO:0005886~plasma membrane | 38 | 0.092758647 | CKB/PRR15/FGFR1/NID2/EFR3B/WSCD1/TTC28/GSPT2/SMPDL3B/SLC46A3/HAS2/EHD2/AMIGO2/IRF6/TMEM238/SAMD12/MMP14/MMP16/LD2/ALDH2/ALDH1A3/EFEMP1/VWDE/SYTL2/TMEM131L/WHRN/GALNT3/CCDC69/HSPB8/GJA1/ANKRD1/GPR137C/ANK3/GRPR/GUCY1B1/FOXA2/HOXB9/IFI16/MACC1 |
| GO:0070062~extracellular exosome | 33 | 0.005937755 | TMEM238/SAMD12/MMP14/MMP16/MXRA7/CSNK1D/  C19orf60/TAOK3/SERPINF1/MET10/MANSC1/PDE1/SAP155/PLAT/XCR1/PTPRE/RBM47/RPL22L1/RNF207/N4BP2/SNORA11D/TF/SCN8A/AVT6/HTRA1/PRICKLE2/ATP10A/STRIP2/PLEKHG1/PIGK/PTPRM/TMEM120B/ABHD17C |
| GO:0005615~extracellular space | 32 | 7.12E-09 | IFIH1/FRG1JP/MXI1/ST14/YCL042W/ZEB1/ARHGAP28/RSRC1/IRX5/EXO1/CNTNAP2/LINC00941/CTS1/AASS/ACE2/MPZL2/LINC01291/TPP1/TMEM45B/H4C6/FGD4/MKC7/SDC2/AXIN2/TINAGL1/P3H1/RAB17/FAD1/SLC1A3/RRI1/SPINT1/SRD1 |
| GO:0005576~extracellular region | 32 | 4.09E-07 | FGFR1/NID2/EFR3B/WSCD1/EHD2/AMIGO2/IRF6/TMEM238/CTS1/AASS/ACE2/MPZL2/PDE1/SAP155/PLAT/XCR1/MKC7/SDC2/AXIN2/TINAGL1/MIG7/ILS1/ZNF165/ZNF165/PRRG4/PAP2/BSC5/SPS18/CMTM3/Blk/Atp1b2/WAR1 |
| GO:0007165~signal transduction | 17 | 0.019919115 | MET10/MANSC1/LINC00261/SER3/JPH1/ACE2/MPZL2/IGFBP3/KRT18P10/ITGB3/AREG/PEAR1/ANXA2R/LAD1/LCN2/LYS1/IRC24 |
| GO:0005887~integral component of plasma membrane | 17 | 0.052840692 | H4C6/FGD4/MKC7/SDC2/ |
| GO:0043547~positive regulation of GTPase activity | 13 | 0.001671087 | MKC7/SDC2/AXIN2/TINAGL1/P3H1/RAB17/FAD1/SLC1A3/RRI1/SPINT1/SRD1/DUR1/TPD52L1 |
| GO:0043066~negative regulation of apoptotic process | 12 | 0.000935313 | MIG7/ILS1/ZNF165/ZNF165/PRRG4/SMN1/TFPI2/AHNAK/H3C10/MND1/FAM78B/ZNF382 |
| GO:0042802~identical protein binding | 11 | 0.053712203 | IFIH1/MXI1/ST14/ZEB1/RSRC1/IRX5/EXO1/CNTNAP2/HAS2/EHD2/SAMD12 |
| GO:0006955~immune response | 10 | 0.006062392 | ZEB1/RSRC1/TMEM238/SAMD12/MMP14/MMP16/MXRA7/CSNK1D/TAOK3/MET10 |
| GO:0010628~positive regulation of gene expression | 9 | 0.00109994 | PLAT/XCR1/PTPRE/RBM47/RPL22L1/IFIH1/FRG1JP/MXI1/ITGB3 |
| GO:0046982~protein heterodimerization activity | 9 | 0.023336447 | PTPRM/EXO1/HAS2/EHD2/AMIGO2/IRF6/TAOK3/SERPINF1/MET10 |
| GO:0007267~cell-cell signaling | 8 | 0.003962885 | MXI1/ST1/PLEKHG1/PIGK/PTPRM/EXO1/HAS2/EHD2/AMIGO2/IRF6/ |
| GO:0000165~MAPK cascade | 8 | 0.004689123 | PIGK/PTPRM/EXO1/HAS2/EHD2/IRF6/SAMD12/MMP14/MMP16/ |
| GO:0006954~inflammatory response | 8 | 0.030214957 | HAS2/EHD2/AMIGO2/SAMD12/MMP14/MMP16/TAOK3/SERPINF1 |
| GO:0008284~positive regulation of cell proliferation | 8 | 0.074977639 | FRG1JP/MXI1/ST1/PLEKHG1/PIGK/TAOK3/SERPINF1/MET10 |
| GO:0016324~apical plasma membrane | 8 | 0.005548599 | ALDH2/ALDH1A3/EFEMP1/ZNF382/P4HA/PAP2/BSC5/SPS18 |
| GO:0016032~viral process | 7 | 0.030835763 | TXNIP/CCDC69/HSPB8/GJA1/IGFBP3/KRT18P10/ITGB3 |
| GO:0042493~response to drug | 7 | 0.033044145 | IGFBP3/KRT18P10/ITGB3/AREG/FAD1/SLC1A3/RRI1 |
| GO:0008283~cell proliferation | 7 | 0.06909926 | MPZL2/FOXA2/HOXB9/MIG7/ILS1/ZNF165/ZNF165 |
